# Impaired perception of surface tilt in progressive supranuclear palsy

**DOI:** 10.1371/journal.pone.0173351

**Published:** 2017-03-07

**Authors:** Marian L. Dale, Fay B. Horak, W. Geoffrey Wright, Bernadette M. Schoneburg, John G. Nutt, Martina Mancini

**Affiliations:** 1 Department of Neurology, Oregon Health & Science University, Portland, Oregon, United States of America; 2 Parkinson’s Disease Research, Education and Clinical Center, P3-PADRECC, Veterans Affairs Portland Healthcare System (VAPORHCS), Portland, Oregon, United States of America; 3 Departments of Physical Therapy and Bioengineering, Temple University, Philadelphia, Pennsylvania, United States of America; Oslo Universitetssykehus, NORWAY

## Abstract

**Introduction:**

Progressive supranuclear palsy (PSP) is characterized by early postural instability and backward falls. The mechanisms underlying backward postural instability in PSP are not understood. The aim of this study was to test the hypothesis that postural instability in PSP is a result of dysfunction in the perception of postural verticality.

**Methods:**

We gathered posturography data on 12 subjects with PSP to compare with 12 subjects with idiopathic Parkinson’s Disease (PD) and 12 healthy subjects. Objective tests of postural impairment included: dynamic sensory perception tests of gravity and of surface oscillations, postural responses to surface perturbations, the sensory organization test of postural sway under altered sensory conditions and limits of stability in stance.

**Results:**

Perception of toes up (but not toes down) surface tilt was reduced in subjects with PSP compared to both control subjects (p≤0.001 standing, p≤0.007 seated) and subjects with PD (p≤0.03 standing, p≤0.04 seated). Subjects with PSP, PD and normal controls accurately perceived the direction of gravity when standing on a tilting surface. Unlike PD and control subjects, subjects with PSP exerted less postural corrective torque in response to toes up surface tilts.

**Discussion:**

Difficulty perceiving backward tilt of the surface or body may account for backward falls and postural impairments in patients with PSP. These observations suggest that abnormal central integration of sensory inputs for perception of body and surface orientation contributes to the pathophysiology of postural instability in PSP.

## Introduction

Falls are a major clinical burden in progressive supranuclear palsy (PSP), a neurodegenerative tauopathy with rapid progression and median survival from five to ten years (Richardson variant) [1–5]. In contrast to idiopathic Parkinson’s Disease (PD), postural instability and backward falls occur early in the clinical course of PSP [5]. Clinical features that may contribute to falls in PSP include vertical supranuclear gaze limitations, rigidity, retrocollis, and cognitive dysfunction [5]. The contribution of specific impairments in postural control in PSP that leads to backward disequilibrium is poorly understood.

Previous studies suggest that peripheral vestibular dysfunction may contribute to postural instability in PSP [5,6]. These studies utilized the sensory organization test (SOT), in which subjects are asked to stand still under a variety of altered visual and/or somatosensory conditions. The SOT condition that best differentiated PSP from PD was the most difficult condition, when both visual and somatosensory inputs were altered [6]. Although abnormalities in this condition of the SOT abnormality are consistent with a “vestibular loss pattern” of dysfunction [6], central multisensory processing deficits cannot be ruled out. Translational vestibulo-ocular reflexes (VORs) are diminished with near target viewing in patients with PSP [5], providing further evidence for vestibular impairment, but the relative contribution of vestibular impairment to postural instability in PSP is unclear. It is unknown whether patients with PSP have abnormal perception of gravitational or surface orientation in space. We hypothesized that dysfunction of the multisensory integration involved in the perception of verticality or surface orientation may be a critical mechanism of postural instability and backward falls in patients with PSP.

Our study specifically compared perception of surface tilt versus the perception of gravitational vertical in PSP when sitting or standing on a tilting surface that oscillates, causing dorsi- and plantar-flexion at the ankle. Automatic postural responses to surface translations or rotations have not been reported for patients with PSP. Based on the tendency of PSP patients to fall backward, we hypothesized that patients with PSP would have deficits recovering from backward body perturbations due to disturbed automatic postural responses to surface translations or rotations.

## Methods

### Subjects

This cross-sectional study recruited 12 patients meeting diagnostic criteria for probable PSP [7], 12 patients with a clinical diagnosis of idiopathic PD, and 12 healthy controls from the Parkinson’s Center of Oregon, Oregon Health and Science University, Portland, Oregon. The subjects with PSP all exhibited a gradually progressive disorder with onset after age 63, vertical supranuclear gaze palsy, postural instability, and no evidence of another explanatory disease, consistent with the NINDS and Society for PSP diagnostic criteria [7]. The PSP and PD subjects were matched by Unified Parkinson’s Disease Rating Scale (UPDRS) III *off* levodopa motor score. The control and PSP subjects were matched by age and sex (see Table 1). All gave informed, written consent to a protocol approved by the Institutional Review Board of Oregon Health and Science University.

**Table 1:** Participant characteristics.

*PSP*	*Age*	*Sex*	*Symptom onset*	*Falls*	*UPDRS III*	*PIGD*	*PSP RS*
PSP01	76	F	2.5	5	33	7	36
PSP02	64	F	4	28	22	6	29
PSP03	63	M	2	0	35	6	43
PSP04	66	M	3	4	53	12	50
PSP05	70	F	6	0	45	11	24
PSP06	70	F	3	1	39	12	27
PSP07	67	M	2	-	31	9	26
PSP08	78	F	1	0	29	6	17
PSP09	71	M	0.6	-	30	5	15
PSP10	63	M	1	1	29	-	30
PSP11	69	F	0.2	-	24	5	9
PSP12	83	M	3	-	43	6	17
***Mean***	***70***		***2*.*6***	***4*.*9***	***34*.*4***	***7*.*7***	***26*.*9***
***STD***	***6*.*3***		***1*.*5***	***9*.*5***	***9*.*1***	***2*.*8***	***11*.*9***
***PD***							
PD01	58	M	5	0	35	5	
PD02	61	M	16	0	53	5	
PD03	71	M	5	0	38	3	
PD04	59	M	18	0	31	5	
PD05	68	F	11	1	21	3	
PD06	76	F	7	-	30	4	
PD07	70	M	13	0	35	6	
PD08	71	F	7	0	35	7	
PD09	72	F	2	-	29	3	
PD10	81	M	4	-	36	6	
PD11	59	F	1	-	30	3	
PD12	67	F	-	-	31	5	
***Mean***	***67*.*8***		***8*.*1***	***0*.*1***	***33*.*7***	***4*.*6***	
***STD***	***7*.*3***		***5*.*6***	***0*.*4***	***7*.*5***	***1*.*4***	

symptom onset = years from onset of symptoms, falls = self-reported in last month, UPDRS III = part 3 of Unified Parkinson’s Disease Rating Scale, PIGD = postural instability gait difficulty sub-score of UPDRS III, PSP RS = PSP Rating Scale, PD = Parkinson’s Disease,— = missing data point

The subjects with PSP and PD did not differ significantly in age or gender, though subjects with PSP had a higher PIGD (postural instability gait difficulty) sub-score and reported more frequent falls. There was minimal difference in pharmaceutical anticholinergic burden between the groups: two subjects with PSP were taking anticholinergic medication at the time of testing (daily diphenhydramine in one case and tolterodine in the other), compared to one PD subject prescribed both tolterodine and darifenacin. One subject with PSP was taking donepezil, whereas no subjects with PD were concomitantly receiving cholinergic augmenting medications. Levodopa equivalent doses (LEDs) of non-levodopa medications (including COMT inhibitors, dopamine agonists, MAO-B inhibitors, and amantadine) averaged 225.7 mg/day in the PD group. Two subjects with PSP were taking amantadine (200 mg LED).

### Protocol

The participants underwent clinical assessment and quantitative balance evaluation. A neurological exam and UPDRS III were administered by a movement disorders specialist prior to the balance assessment. Subjects with PD were tested in the *off* levodopa state after a 12-hour medication washout period.

Balance measurements were obtained in seated and standing conditions on the NeuroCom Balance Master Clinical Research System platform (Neurocom International, Inc, Clackamas, Or). When standing, patients’ feet were carefully aligned over a defined axis on the force plate, which is referenced to the four vertical force transducers mounted beneath a supporting center plate. The force plate recorded the vertical forces that were used to calculate the center of pressure (CoP) [8]. Data were collected at 100Hz sampling frequency. In addition, participants were equipped with 3 Opal (APDM, Inc) inertial sensors placed on the posterior trunk at L5 level, on the right ankle and on the forehead to measure body tilt. A fourth inertial sensor was placed on a 30cm wooden rod used for the subject to indicate their perception of verticality.

**Fig 1:**
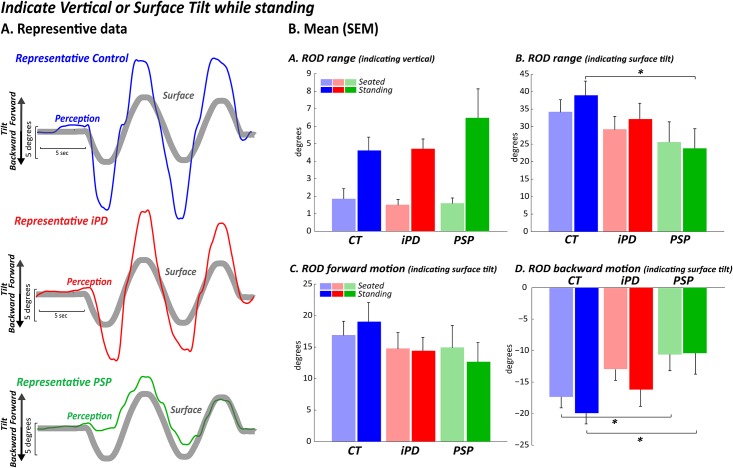
Perception of gravitational vertical and support surface orientation during platform oscillations. Using an instrumented rod, healthy control subjects and iPD subjects similarly overestimated surface orientation, consistent with previous studies. In contrast, subjects with PSP showed reduced perception of surface movement and nearly absent perception of backward surface tilt. AP: anterior-posterior A) Rod range when indicating vertical in seated and standing conditions (difference from vertical) B) Rod range when indicating surface tilt in seated and standing conditions C) Rod maximum forward motion when indicating surface tilt in seated and standing conditions D) Rod minimum backward motion when indicating surface tilt in seated and standing conditions.

The postural stability tasks and corresponding metrics are described below:

1. Perception of gravitational vertical and support surface orientation during platform oscillations
  Subjects had their eyes closed when standing on the platform that was tilting backward and forward. The surface tilt was a slow sinusoidal rotation at 0.025Hz. The surface tilted ±5° centered about the ankle joints. A similar slow oscillation paradigm designed to test frequencies below perception of the vestibular semicircular canal system has been described by Azulay et al and others [9,10]. Subjects were instructed 1) to align the instrumented rod parallel with the earth-fixed vertical (measuring the ability to sense gravitational vertical), and 2) to hold the rod perpendicular to the tilting support surface, using both hands (measuring the ability to sense the surface orientation). The same conditions were repeated with the subjects sitting on a stable, earth-fixed chair with the subjects’ feet placed firmly on the tilting surface. For a representative image of the set-up, please see Fig 1 of Wright and Horak’s previously published manuscript [11]. This handheld rod technique has been shown to be highly precise (1°±0.7°) when used to indicate gravitational vertical and perceived postural vertical [12–14], and directionally unbiased in healthy individuals when indicating surface orientation, despite overestimation of the amplitude of the surface tilt amplitude [12,14]. In order to avoid any possible pendular effect predisposing the rod toward gravitational vertical, the rod was designed to be lightweight (<200 grams) and equally balanced in the hands of subjects when held at midpoint [11]. Each trial lasted 25s, which included 5s of quiet stance, followed by 20s of sinusoidal surface tilt, and we obtained mean values over 3 trials. From the inertial sensor on the rod we calculated the rod vertical tilt (for the gravitational vertical), and the rod forward and backward range (for the surface tilt).
2. Postural Motor Control: Forward Platform Translation assessed the ability of the automatic postural motor system to quickly recover following unexpected external disturbances. Forward translations of the support surface that moved the body backward were delivered without warning at 15 cm/sec 3 times. We quantified the strength of response to the perturbations by calculating the Peak CoP during the platform translation and the Shift of the CoP position from baseline.
3. Toes-up Platform Rotation assessed the postural response to backward body tilt. A platform tilt of 15 degrees was delivered 3 times. Each trial lasted 15s, which included 5s of quiet stance at the beginning before the platform rotation. From the recorded CoP we quantified the *Peak CoP* from baseline, indicative of the plantar flexion applied to maintain balance.

### Statistical analysis

The normal distribution of data was confirmed with Kolmogorov-Smirnov tests. For our primary outcome measures a one-way ANOVA test was used to determine whether differences existed among the 3 subject groups on each measure. When a significant difference was found (p≤0.05), a post hoc analysis was performed using Bonferroni adjustment (P ≤0.01 for 3 pair-wise comparisons) to test which group (control, PD, PSP) differed from each other. Because the PIGD scores in the PSP group were higher than in the PD group, we tested for an association between PIGD and our outcome measures using Spearman’s correlation coefficient as an exploratory analysis on the metrics in which we found significant group effects. All the analyses were performed using Matlab (MathWorks, Natick, Massachusetts) and SPSS (IBM SPSS Statistics 22).

## Results

### Subjects with PSP showed a reduced perception of toes-up platform tilt (backward surface tilt) compared to control subjects and PD subjects

A significant group effect was found for the rod range with subjects standing while indicating surface tilt, F_2,33_ = 3.5, p≤0.04, (Fig 1B, darker colors). Post hoc analysis revealed that subjects with PSP moved the instrumented rod to indicate surface tilt significantly less than the healthy subjects (p≤0.01), and only slightly less (p≤0.05) compared to the subjects with PD; subjects with PD were similar to healthy controls (p≤0.1). Specifically, subjects with PSP showed a reduction in rod movement past midline when the surface tilted backward (group effect F_2,33_ = 6.3, p≤0.007) compared to the healthy subjects (p≤0.001) and to the PD subjects (p≤0.03). Subjects with PD were similar for backward tilt to healthy controls (p≤0.1), represented in Fig 1D. However, when tilted forward, no differences across groups were detected (F_2,33_ = 1.4, p≤0.2), as represented in Fig 1C, darker colors.

Similar to standing, a significant group effect for backward tilt (F_2,33_ = 4.2, p≤0.02) was found when subjects were seated (lighter colors) with their feet on the tilting surface. Post hoc analysis indicated that subjects with PSP tilted the rod significantly less compared to healthy subjects (p≤0.007) and slightly less compared to PD (p≤0.04 not significant due to Bonferroni correction). Subjects with PD also tilted the rod slightly less compared to healthy subjects (p≤0.04, not significant due to Bonferroni correction). When tilted forward, no difference across groups were detected (F_2,33_ = 0.37, p≤0.69). However, while sitting, no significant group effect was found in the total rod range (F_2,33_ = 3.38, p≤0.05).

### Subjects with PSP correctly perceived the direction of gravity when standing on an oscillating platform

No group effect was found in the rod range while subjects tried to maintain the rod upright to indicate verticality (Fig 1A) for both the standing condition (F_2,33_ = 2.4, p≤0.1) or the sitting condition with the feet placed on the tilting platform (F_2,33_ = 0.12, p≤0.9).

### When displaced backward, subjects with PSP remained backward

**Fig 2:**
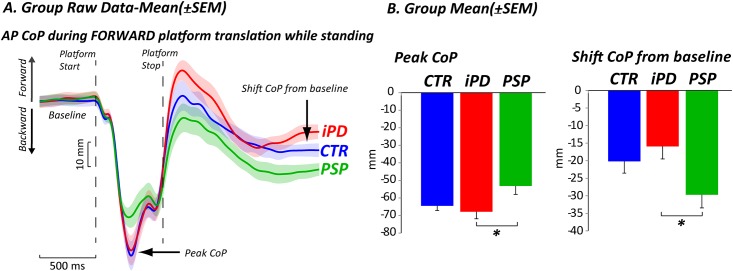
Postural motor control: Forward platform translation. CoP displacement in response to forward platform translations. A) Group raw data mean±SEM B) Group mean±SEM. CoP: center of pressure, CoM: center of mass; AP: anterior-posterior.

During the forward translation of the platform, which moved the body backward, there was a significant group effect on the backward peak of the center of pressure (CoP) (F_2,33_ = 3.8, p≤0.03). Specifically, subjects with PSP displaced their CoP significantly less than PD subjects (p≤0.006) and slightly less than healthy subjects (p≤0.02, not significant due to Bonferroni correction); while subjects with PD were similar to healthy subjects (p≤0.3) (See Fig 2A for raw data and 2B for means). When the platform returned to baseline after the perturbation, a significant group effect was found in the final CoP position (shift from baseline), F_2,33_ = 3.7, p≤0.03. The CoP of subjects with PSP remained more posterior after the platform shifted back to the initial position compared to subjects with PD (p≤0.01), while only a slight difference was found compared to healthy subjects (p≤0.04 not significant due to the Bonferroni correction); again, subjects with PD were similar to healthy subjects (p≤0.2).

### Subjects with PSP exerted less postural corrective torque

**Fig 3:**
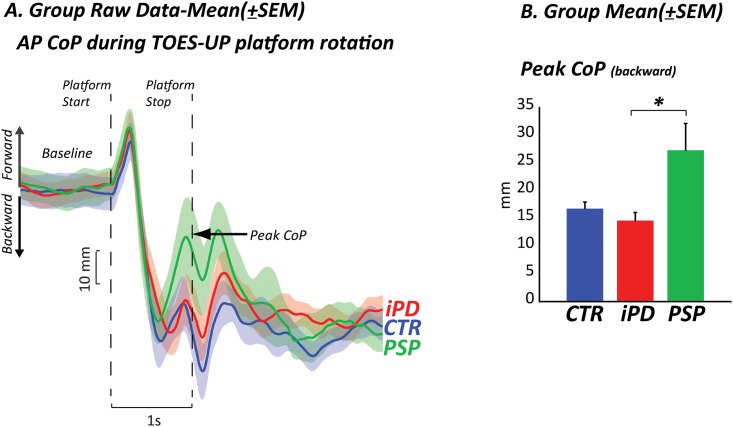
Postural motor control: Toes-up platform rotation. CoP: center of pressure displacement A) Group raw data mean±SEM and B) Group mean±SEM, CoM: center of mass.

When the body was displaced backward by toes-up platform rotation, the CoP shifted quickly backward and stabilized before the end of the perturbation (Fig 3A). A significant group effect was found in the backward position of the CoP at the end of the perturbation (F_2,33_ = 5.87, p≤0.0068), Fig 3B. In this case, subjects with PSP exerted a significantly larger destabilizing plantar-flexion torque (as evidenced by forward CoP displacement) than subjects with PD (p≤0.008), and only slightly larger compared to healthy subjects (p≤0.04, not significant due to Bonferroni correction).

### Sensory organnization tests and limits of stability

The sensory organization (SOT) and limits of stability (LOS) tests were consistent with previous studies in PSP [6, 15] and are therefore presented in supplementary material.

## Discussion

Our findings are consistent with the concept of disturbed central somatosensory integration contributing to backward postural instability in patients with PSP [16]. Unlike patients with idiopathic PD or age-matched control subjects, patients with PSP showed a specific inability to detect when the surface under their feet tilted in a toes-up direction. Furthermore, patients with PSP demonstrate reduced and inappropriate postural motor corrections that contributed to backward body disequilibrium. In fact, when displaced backward with surface translations, patients with PSP remained backward, and when tipped backward, their center of pressure (CoP) shifts forward, consistent with active plantar-flexion at the ankles, and resulting in additional, backward lean. In contrast, subjects with PD and healthy controls did not remain backward after translations and their CoP displacement did not suggest abnormal ankle plantar-flexion torque in response to toes-up platform rotation. Although we did not record EMG activity, this excessive forward CoP displacement to toes up surface tilts has been shown to be associated with activation of the stretched gastrocnemius and soleus muscles, which is normally inhibited in healthy subjects [17].

Furthermore, the PSP subjects’ pattern of plantar flexion in response to a backward tilt is directly contrary to the response of neurologically normal aged adults. Blaszczyk et al established that the elderly dorsiflex as protective strategy against a diminished posterior stability margin [18,19]. The ankle dorsiflexsion results in a mild anterior shift of the center of gravity away from the maximum voluntary excursion [18,19].

Our results suggest that proprioceptive sensory information is not properly integrated with other sensory information to maintain upright posture in PSP. We propose that the deficient vestibular output previously noted in PSP is possibly a result of abnormal central sensory integration, rather than being solely due to a primary vestibular deficit.

Further support for more than just a vestibular deficit comes from the SOT. SOT conditions that use an unstable support surface (i.e. sway-referenced surface) were all significantly worse in the PSP group (eyes open and closed), which suggests dysfunction in multisensory selection and integration. Further, if this were a primary vestibular deficit, then we would expect to see difficulty in perceiving gravitational vertical, which was not the case. Moreover, central vestibular lesions of the parietal insular cortex (the so-called vestibular cortex) are associated with lateral, not backward, postural instability and affect perception of visual, but not postural, surface orientation [20].

Abnormal perception of surface tilt in PSP patients preferentially affected the posterior (toes up tilt) direction, with relatively intact perception of anterior (toes down) tilt. This finding is of particular interest since patients with PSP preferentially fall backward. The correspondence between perceptual asymmetry and posterior falls has been reported in other populations. There is a correlation between backward falls and the abnormal perception of postural and haptic (kinesthetic) vertical in elderly with backward disequilibrium who are otherwise healthy [21].

Our study is the first to demonstrate inappropriate adaptive postural motor control with excessive forward CoP displacement in response to toes-up surface tilts in PSP. There are several reasons why normal compensatory mechanisms for backward excursion may fail in subjects with PSP. Even though their maximum backward voluntary excursion is smaller, they may still require more time to complete a recovery program in response to backward tilt. Poor quality sensory information caused by increased body sway [22] may slow the postural response. Impaired internal representation of vertical posture independent of the gravitational field (body schema) also contributes to the slowed postural response [23, 24]. Cognitive dysfunction [25] may slow detection of the perturbation of the platform tilt in subjects with PSP and force an inappropriately calibrated and excessive anterior CoP response via gastrocnemius and soleus activation, resulting in a counterproductive, backward body push.

Limitations of our study include small sample size, poor match in PIGD between subjects with PSP and PD, and the possibility of confounding due to other sensory abnormalities. Although PIGD scores were higher in subjects with PSP compared to controls, we do not believe that our outcome measures were influenced by PIGD severity; a Spearman’s correlation between the PIGD scores and outcome measures in PD and PSP was insignificant (p>0.05). Regarding sensory abnormalities, none of the subjects complained of neuropathic sensory disturbances in their feet, but toe proprioception was not directly measured. However, it is unlikely that a peripheral neuropathy would result in a directionality of postural disturbance, and thus it should not bias the results. In fact, in Masdeu and Gorelick’s thalamic astasia study, equal numbers of patients showed proprioceptive loss as those with intact proprioception, suggesting that the imbalance was not necessarily due to proprioceptive losses [26]. Similarly, though it was not measured, diminished visual acuity should not influence the directionality of our findings. Because sensory perception testing was consistently performed with eyes closed, the lack of specific visual field and vestibulo-ocular reflex testing in this study should not have biased the results.

Future dynamic perception studies with simultaneous inclusion of cervical vestibular evoked myogenic potentials would help clarify the contribution of vestibular otolith function and potential resultant vestibulospinal tract abnormalities to postural instability in PSP. Future studies should also examine the cognitive contribution to inappropriate postural motor control in PSP. Incorporating advanced imaging techniques such as regional cerebral and thalamic acetylcholinesterase PET or FDG-PET may provide more generalizable evidence for central sensory integration deficits in PSP.

## Appendix

Please see supplementary material for sensory organization test (SOT) and limits of stability (LOS) figures.

## Supporting information

S1 Fig Sensory organization test. Group mean and SE equilibrium scores in controls (blue), PD (red) and PSP (green) groups. * are differences between control and PSP subjects; Six sensory conditions: C1 = eyes open, C2 = eyes closed, C3 = sway-referenced visual surround, C4 = sway-referenced surface with eyes open, C5 = sway-referenced surface with eyes closed, C6 = sway-referenced surface and visual surround. Consistent with previously reported findings, subjects with PSP performed significantly worse compared to subjects with PD subjects and healthy subjects in conditions 4 (PSPvsPD p≤0.004, PSPvsCTR p≤0.005), 5 (PSPvsPD p≤0.0001, PSPvsCTR p≤0.0001), and 6 (PSPvsPD p≤0.0001, PSPvsCTR p≤0.0001).
(TIF)

S2 Fig Limits of stability test. Group mean maximum CoP excursion during the limits of stability test in control, PD, and PSP subjects, as % of target point. FWD: forward, direction R_FWD: right forward direction, RIGHT: right direction, R_BKW: right backward direction, BKW: backward direction, L_BKW: left backward direction, LEFT: left direction, and L_FWD: left forward direction. Consistent with previous findings, subjects with PSP showed significantly smaller limits of stability in all directions compared to healthy subjects (p≤0.01). Subjects with PSP showed similar limits of stability compared to subjects with PD with two exceptions for the right backward (p≤0.01) and backward directions (p≤0.009). The values for the leftward direction were almost identical in subjects with PSP and PD, consistent with previous findings.
(TIF)
