# Supplementary figures and images for: Impaired perception of surface tilt in progressive supranuclear palsy
Source: PLoS One. 2017 Mar 7;12(3):e0173351. doi: 10.1371/journal.pone.0173351 (PMC5340402; doi:10.1371/journal.pone.0173351)

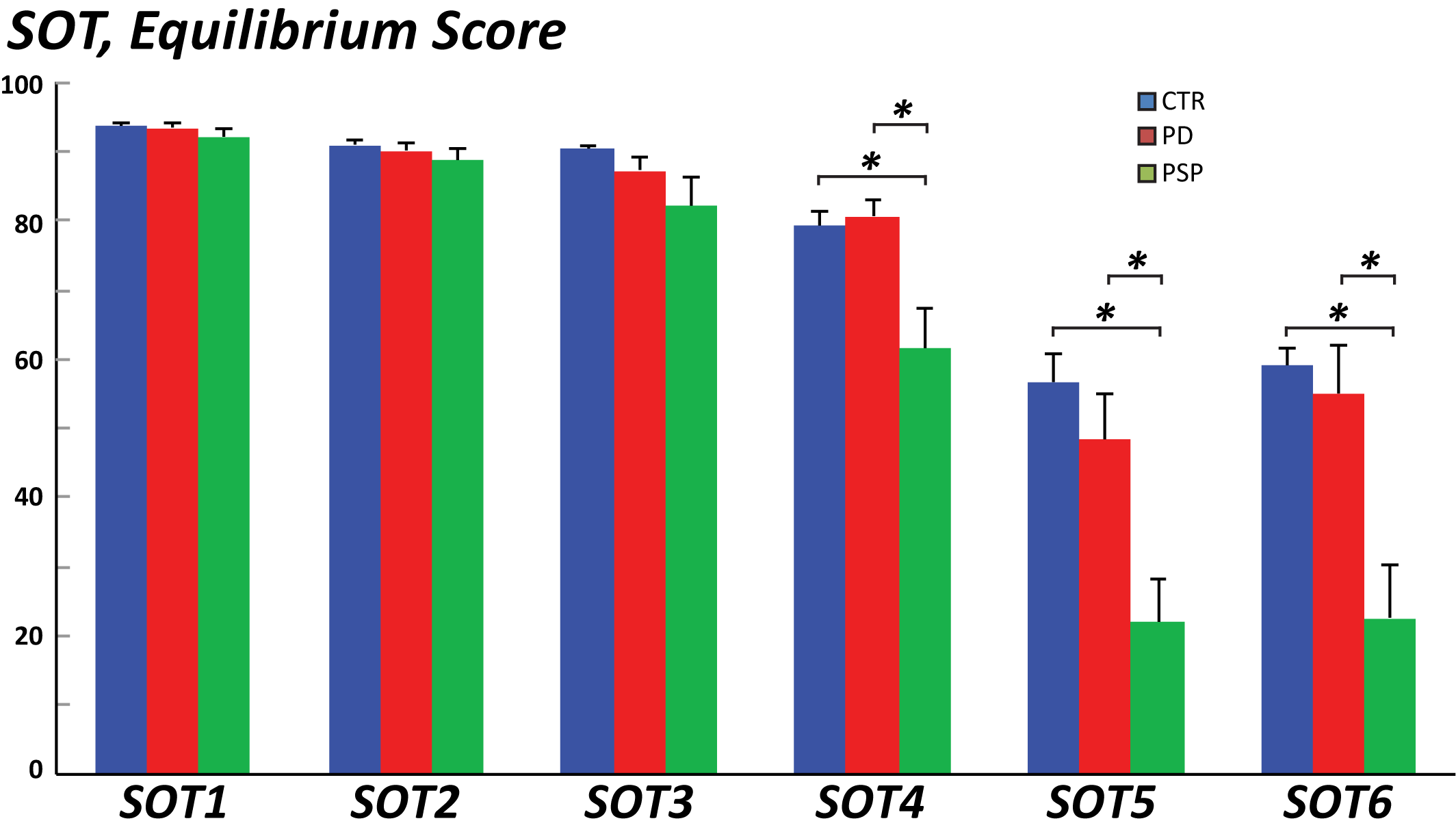

Supplement: S1 Fig — Group mean and SE equilibrium scores in controls (blue), PD (red) and PSP (green) groups. * are differences between control and PSP subjects; Six sensory conditions: C1 = eyes open, C2 = eyes closed, C3 = sway-referenced visual surround, C4 = sway-referenced surface with eyes open, C5 = sway-referenced surface with eyes closed, C6 = sway-referenced surface and visual surround. Consistent with previously reported findings, subjects with PSP performed significantly worse compared to subjects with PD subjects and healthy subjects in conditions 4 (PSPvsPD p≤0.004, PSPvsCTR p≤0.005), 5 (PSPvsPD p≤0.0001, PSPvsCTR p≤0.0001), and 6 (PSPvsPD p≤0.0001, PSPvsCTR p≤0.0001). (TIF) [file pone.0173351.s001.tif]

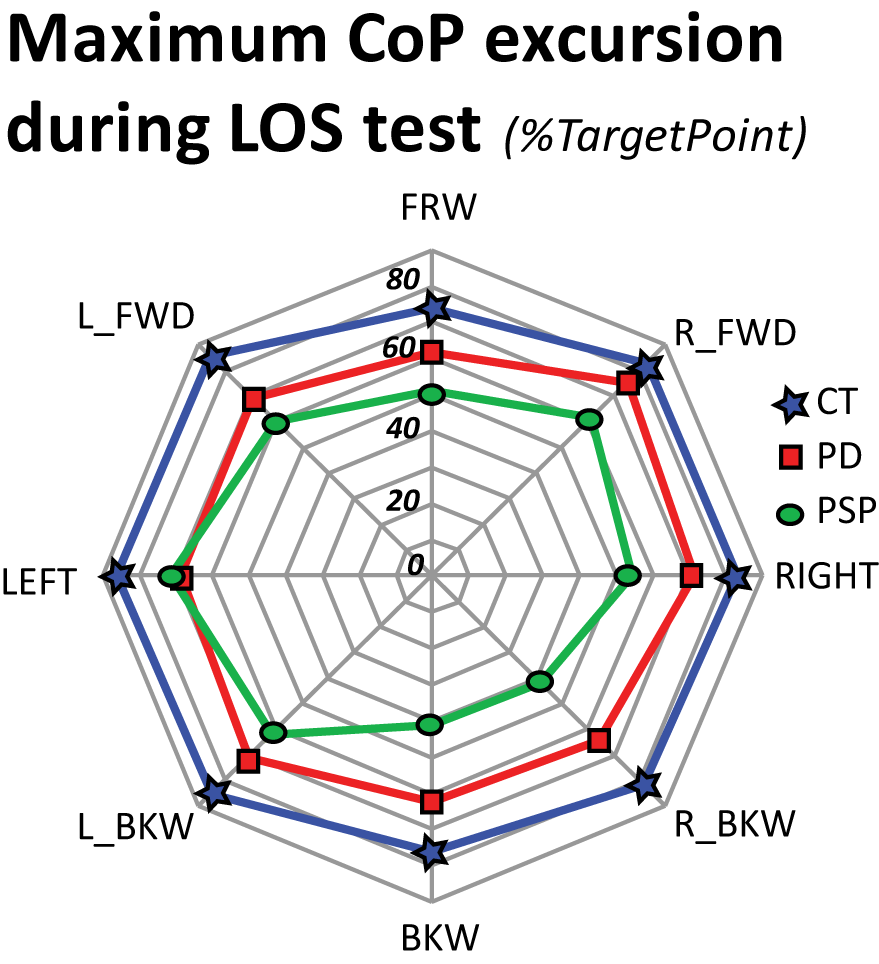

Supplement: S2 Fig — Group mean maximum CoP excursion during the limits of stability test in control, PD, and PSP subjects, as % of target point. FWD: forward, direction R_FWD: right forward direction, RIGHT: right direction, R_BKW: right backward direction, BKW: backward direction, L_BKW: left backward direction, LEFT: left direction, and L_FWD: left forward direction. Consistent with previous findings, subjects with PSP showed significantly smaller limits of stability in all directions compared to healthy subjects (p≤0.01). Subjects with PSP showed similar limits of stability compared to subjects with PD with two exceptions for the right backward (p≤0.01) and backward directions (p≤0.009). The values for the leftward direction were almost identical in subjects with PSP and PD, consistent with previous findings. (TIF) [file pone.0173351.s002.tif]
